# Vitamin D and Human Skeletal Muscle

**DOI:** 10.1111/j.1600-0838.2009.01016.x

**Published:** 2010-04

**Authors:** B Hamilton

**Affiliations:** ASPETAR, Qatar Orthopaedic and Sports Medicine HospitalDoha, Qatar

**Keywords:** IGF-1, injury, deficiency

## Abstract

Vitamin D deficiency is an increasingly described phenomenon worldwide, with well-known impacts on calcium metabolism and bone health. Vitamin D has also been associated with chronic health problems such as bowel and colonic cancer, arthritis, diabetes and cardiovascular disease. In recent decades, there has been increased awareness of the impact of vitamin D on muscle morphology and function, but this is not well recognized in the Sports Medicine literature. In the early 20th century, athletes and coaches felt that ultraviolet rays had a positive impact on athletic performance, and increasingly, evidence is accumulating to support this view. Both cross-sectional and longitudinal studies allude to a functional role for vitamin D in muscle and more recently the discovery of the vitamin D receptor in muscle tissue provides a mechanistic understanding of the function of vitamin D within muscle. The identification of broad genomic and non-genomic roles for vitamin D within skeletal muscle has highlighted the potential impact vitamin D deficiency may have on both underperformance and the risk of injury in athletes. This review describes the current understanding of the role vitamin D plays within skeletal muscle tissue.

Vitamin D is a secosteroid produced in the skin, under the influence of ultraviolet-B (UVB) radiation converting 7-dehydrocholesterol to pre-vitamin D_3_. In the dermis, pre-vitamin D_3_ is rapidly converted to vitamin D_3_ (cholecalciferol), before its subsequent conversion to 25-hydroxy vitamin D (25(OH)D) in the liver. Further hydroxylation of 25-hydroxy vitamin D to its active form, 1,25 hydroxy vitamin D (1,25(OH)_2_D), occurs in the kidney (Holick, 2007). Lesser quantities are also found in the diet in the form of vitamin D_2_ (ergocalciferol), which undergoes the same hydroxylation process. Vitamin D is transported in the blood, bound to vitamin D binding protein.

Vitamin D is well recognized for its role in calcium and phosphorus homeostasis. In conjunction with parathyroid hormone (PTH), vitamin D plays a critical role in calcium homeostasis (Cashman, 2007), and as a result, vitamin D deficiency has been implicated in osteoporotic and stress-related fractures (Ruohola et al., 2006; Lappe et al., 2007, 2008). Less well recognized are its genomic and non-genomic roles in other tissues (Holick, 2006), despite cross-sectional data linking vitamin D deficiency to bowel and colonic cancer, arthritis, diabetes and cardiovascular disease (Pani et al., 2000; Hypponen et al., 2001, 2008; Dietrich et al., 2004; Holick, 2004, 2006, 2007; Garland et al., 2006; Giovannucci et al., 2006; Lappe et al., 2007; Pittas et al., 2007).

Both the definition of vitamin D deficiency and what constitutes an appropriate supplementation strategy continue to be debated (Malabanan et al., 1998; Dawson-Hughes et al., 2005; Bischoff-Ferrari et al., 2006; Bischoff-Ferrari & Dawson-Hughes, 2007; Hansen et al., 2008;). While 1,25(OH)_2_D is the active form of vitamin D, 25(OH)D is the appropriate index for estimating whole-body vitamin D levels. This is the result of both its substantially higher concentration and the fact that even with a marked vitamin D deficiency, elevated PTH levels will maintain the conversion of 25(OH)D to 1,25(OH)_2_D, thereby sustaining 1,25(OH)_2_D levels within normal ranges, despite low reserves (Holick, 2004). It is generally accepted that 25(OH)D levels of 20–30 ng/mL (ng/mL × 2.5=nmol/L) represent insufficiency, while levels below 20 and 10 ng/mL represent deficiency and severe deficiency, respectively (Holick, 2007). Recent evidence suggests that inter-laboratory variability in 25(OH)D assessment may also complicate the interpretation of vitamin D deficiency (Binkley et al., 2004).

Notwithstanding these issues, vitamin D deficiency is increasingly being recognized as a worldwide epidemic (Nowson & Margerison, 2002; Gordon et al., 2004; Hashemipour et al., 2004; Andersen et al., 2005; Rockell et al., 2005; Binkley et al., 2007; Hannan et al., 2008;). With the most common cause of vitamin D deficiency being inadequate sunlight exposure, it is not surprising that higher latitude countries have a high incidence of deficiency (Andersen et al., 2005). However, despite its high sunlight hours, vitamin D deficiency is well recognized in Middle Eastern women (Fonseca et al., 1984; Hatun et al., 2005; Allali et al., 2006;), and more recently in inner city young adults in America (Gordon et al., 2004), elite gymnasts in Australia (Lovell, 2008), young Hawaiian skateboarders (Binkley et al., 2007), and adolescent girls in England (Ward et al., 2009). Vitamin D deficiency may have significant long-term health impacts (Holick, 2004; Giovannucci et al., 2006;), but it is also possible that a deficiency will result in more immediate effects on musculo-skeletal health, with an increased risk of injuries such as stress fractures (Ruohola et al., 2006; Lappe et al., 2007, 2008). While there is no evidence to support or refute the possibility that vitamin D deficiency will affect the injury profile of tissues such as muscle, the evidence presented herein suggests that further research in this area is required. Furthermore, little is known of any performance impact of vitamin D deficiency; however, some authors suspect it may be a marked impediment to performance when not available in adequate levels (Cannell et al., 2009). Indeed, in the early part of the 20th century, athletes were allegedly using UVB rays as an ergogenic aid (Hoberman, 1992; Cannell et al., 2009;) and research over that period suggested that both cardiovascular fitness and muscular endurance were enhanced with exposure to ultraviolet radiation (Allen & Cureton, 1945). While vitamin D deficiency has long been associated with muscle weakness (Floyd et al., 1974; Irani, 1976; Russell, 1994; Ceglia, 2008;), until recently no specific etiological mechanism had been described. Over the last 30 years, an independent mechanism for vitamin D and muscle function has slowly been unraveled. Subsequently, while limited, there is evidence from a range of sources relating vitamin D deficiency to suboptimal muscle function. This review aims to describe the available evidence for a role of vitamin D in muscle function, and thereby its potential impact on the athletic individual.

## Myopathy associated with vitamin D deficiency

Myopathy associated with vitamin D deficient osteomalacia has been recognized for many years, presenting predominantly as a proximal muscle weakness or difficulty in walking upstairs (Floyd et al., 1974; Irani, 1976; Russell, 1994; Ceglia, 2008;). Traditionally, it was felt that this myopathic presentation was secondary to osteomalacia and disuse, rather than a direct effect of vitamin D on muscle; however, the increased mechanistic understanding of vitamin D challenges this presumption (Glerup et al., 2000). As early as 1974, authors had illustrated electromyographic changes in patients with muscle weakness associated with osteomalacia (Floyd et al., 1974), which improved with vitamin D supplementation (Irani, 1976). Subsequently, numerous reports attest to the reversibility of the myopathy associated with vitamin D deficiency (Rimaniol et al., 1994; Russell, 1994; Ziambaras & Dagogo-Jack, 1997; Mingrone et al., 1999; Prabhala et al., 2000;).

Glerup et al. (2000) examined the impact of vitamin D supplementation on vitamin D-associated myopathy. In a preliminary study, a small group (*n*=8) of elderly men and women (mean age 63.1±5.3 years) with known osteomalacia had muscle strength assessed using an isokinetic dynamometer before and after 3 months of treatment with alfacalcidol, ergocalciferol and calcium. They found that over 3 months, muscle power increased significantly in all the muscle groups assessed, with a mean improvement of 24.8±8.0%. Subsequently, they compared a group of vitamin D-deficient Arab women with a control group of Danish women with normal levels of vitamin D. At baseline, quadriceps maximum voluntary contraction (MVC), as well as electrically stimulated twitch [single twitch, maximum production rate (MPR) and maximal relaxation rate (MRR)], were all significantly lower in the Arab women. Three months of vitamin D supplementation, without strength training, increased vitamin D levels and normalized PTH levels in the Arab women, with a corresponding trend toward normalization of the MVC, MPR and MRR. Multivariate regression analysis revealed that only 25(OH)D was significantly associated with MVC. Given that there was no correlation between muscle power and markers of osteomalacia, the authors concluded that normal levels of 25(OH)D are necessary for maintaining adequate muscle function.

## Morphology

Early biopsy studies (Floyd et al., 1974; Irani, 1976;), and subsequent case reports of the muscle weakness associated with osteomalacia (Russell, 1994; Ziambaras & Dagogo-Jack, 1997;), have revealed either non-specific or type II muscle fiber atrophy. Sato et al. (2005) were the first to assess the impact of vitamin D supplementation on muscle histopathology. They biopsied the non-hemiplegic vastus lateralis of 85 vitamin D-deficient elderly stroke patients, before and after a 2-year supplementation period with either placebo or vitamin D_2_. At baseline they found a normal range of type I fibers, but a reduced proportion and diameter of type II muscle fibers. At the 2-year follow-up, the placebo group showed a further reduction in type II muscle fiber diameter, while in the vitamin D_2_-supplemented group the relative content and mean diameter of type II fibers increased, with the fiber size correlating with 25(OH)D levels (Sato et al., 2005).

## Age-related changes in muscle function

It is well recognized that muscle strength declines with age, due to a number of contributory factors (Iannuzzi-Sucich et al., 2002). However, the role of vitamin D in any age-related strength decline continues to be debated (Janssen et al., 2002; Latham et al., 2003a,b;), with the majority of studies being cross-sectional in design (Bischoff et al., 1999; Mowe et al., 1999; Bischoff-Ferrari et al., 2004a,c; Houston et al., 2007; Wicherts et al., 2007;). These studies appear to show a relationship between 25(OH)D levels and various measures of changes in muscle strength and function with aging (Bischoff et al., 1999; Mowe et al., 1999; Bischoff-Ferrari et al., 2004a,c; Houston et al., 2007; Wicherts et al., 2007;). By contrast, a review of randomized-controlled trials investigating vitamin D and/or calcium supplementation concluded that while a combination of calcium and vitamin D may improve physical function and reduce falls, there was no evidence that vitamin D alone improved the strength or the physical function of elderly people (Latham et al., 2003a,b;). Increasingly, however, well-controlled and designed studies support a role for vitamin D in moderating the age-related decline in muscle function (Bischoff et al., 2003; Visser et al., 2003; Gerdhem et al., 2005;), with a potential mechanism described recently (Bischoff-Ferrari et al., 2004a,c; Roth et al., 2004;).

For example, Visser et al. (2003) prospectively investigated the impact of low 25(OH)D and high serum PTH in 1008 men and women aged over 65 years (mean 74 years) and found that individuals with a lower 25(OH)D and/or higher PTH levels were significantly more likely to lose grip strength and muscle mass. They also found 25(OH)D levels of 30 ng/mL to be a threshold for optimal muscle function. In the same year, Bischoff et al (2003) performed a 12-week double-blind, randomized-controlled trial utilizing vitamin D and calcium vs calcium supplementation alone. They reported a significant improvement in knee flexor and extensor strength, grip strength and functional (timed up and go) testing, in their elderly group following vitamin D and calcium, vs calcium supplementation alone. Further, Gerdhem et al. (2005), in a 3-year study of 986 Swedish 75-year-old women, found that reduced 25(OH)D levels correlated with reduced gait speed, reduced knee flexor and extensor strength and increased risk of falls. Similarly, Broe et al. (2007), in a 5-month prospective randomized-controlled trial, illustrated a dose-dependent (800 IU/day) impact of vitamin D supplementation on significantly reducing falls in the elderly. Several other prospective studies have reported similar functional benefits (Verhaar et al., 2000; Dhesi et al., 2004; Sato et al., 2005; Bunout et al., 2006;) and a meta-analysis of studies has confirmed the benefit of vitamin D supplementation on fall prevention (Bischoff-Ferrari et al., 2004b).

By contrast, in a large prospective randomized-controlled trial, Latham et al. (2003a,b); assessed the relative benefits of home resistance exercise or a single high dose of vitamin D, on self-reported physical health, risk of falls and functional performance at 3 and 6 months. Despite increasing the serum 25(OH)D level in those individuals treated, they found no significant impact of 25(OH)D on any of the functional outcome parameters (Latham et al., 2003a,b;).

In an effort to evaluate a potential mechanism of vitamin D-associated changes in muscle morphology and function with age, the expression of the vitamin D Receptor (VDR) was assessed in the gluteus medius and transversospinalis muscles of female patients undergoing hip arthroplasty and spinal operations, respectively (Bischoff-Ferrari et al., 2004a,c;). The authors found that VDR expression decreased with age and that VDR expression was unaffected by either 25(OH)D or 1,25(OH)_2_D levels. The authors suggested that age-related decline in muscle strength may be related to reduced VDR expression. Furthermore, VDR polymorphisms may also result in variable susceptibility to age-related sarcopenia (Roth et al., 2004).

By far the majority of vitamin D studies have been performed on the elderly population, who are prone to sarcopenia, and as a result there is limited evidence for the impact of vitamin D on healthy young individuals. However, El-Hajj et al. (2006) reported a 1-year prospective double-blind, placebo-controlled trial of low- and high-dose vitamin D_3_ in 179 adolescent Lebanese girls. In vitamin D-supplemented individuals they found increased lean mass, bone area and bone mass, particularly in pre-menarchal girls, but found no increase in grip strength. Furthermore, there were no significant findings regarding 25(OH)D and muscle mass or grip strength in a similar cohort of male adolescents (El-Hajj et al., 2006). By contrast, a recent study of 99 post-menarchal adolescent girls in England found a positive relationship between serum 25(OH)D level and jump height, jump velocity and power (Ward et al., 2009).

## Vitamin D and muscle function: mechanistic considerations

### The VDR

Attempts to identify an independent role for vitamin D on skeletal muscle began in earnest in 1975 when 25(OH)D, presumably via a specific receptor, was proposed to directly stimulate the synthesis of protein, ATP and inorganic phosphate in the rat diaphragm muscle (Birge & Haddad, 1975). However, there continued to be some debate as to whether or not vitamin D has a truly independent effect on muscle. Wassner et al. (1983) discredited the aforementioned study, and concluded that vitamin D has no direct effect on muscle, rather its impact was felt to be indirect, via calcium and insulin (Wassner et al., 1983). This debate was settled in 1985 when a VDR was recognized within cultured rat myoblast cells, thereby showing that muscle is a direct target organ for 1,25(OH)_2_D (Simpson et al., 1985). The VDR has subsequently been described in tissues such as smooth muscle, heart muscle, liver, lung, colon, gonads and skin (Pfeifer et al., 2002; Nibbelink et al., 2007;), and was recently isolated from human skeletal muscle (Bischoff et al., 2001; Bischoff-Ferrari et al., 2004a,c;).

1,25(OH)_2_D receptors have been characterized as members of the steroid hormone super-family, acting as a hormone-inducible transcription factor (Peng et al., 2004; Liao et al., 2008;). Furthermore, VDR have been shown to have various genetic polymorphisms, which may affect their function within skeletal muscle (Geusens et al., 1997; Grundberg et al., 2004; Hopkinson et al., 2008;). In combination with co-factors “retinoid × receptor” and “Steroid Receptor Coactivator 3” (SRC), the VDR: 1,25(OH)_2_D complex modulates gene expression of a number of proteins, via binding to specific target gene promoter regions, known as “vitamin D response elements” (VDRE) (Peng et al., 2004; Liao et al., 2008;). This may include both proteins with roles in calcium metabolism such as calbindin (Fleet, 2004), but also proteins not directly related to calcium metabolism such as insulin-like growth factor binding protein 3 (IGFBP-3) (Peng et al., 2004).

To assess the role of the VDR in the skeletal muscle of mice, a generation of VDR gene-deleted mice and myoblast cell lines were examined (Endo et al., 2003). In order to exclude the impact of secondary metabolic abnormalities such as hypocalcemia, gene-deplete mice were assessed at 3 weeks, before weaning and development of secondary metabolic problems. Analysis revealed that VDR null mice had fiber sizes in the quadriceps and other muscle groups 20% smaller than VDR-replete mice and that this trend progressed as secondary metabolic factors developed from 3 weeks of age. Furthermore, VDR null mice exhibited increased expression of myogenic transcription factors myf5, E2A and myogenin compared with normal mice along with inappropriate expression of embryonic and neonatal type myosin heavy chain (Endo et al., 2003). These findings support a direct role for 1,25(OH)_2_D and the VDR in both the metabolic processes and the transcriptional regulation of skeletal muscle. Subsequently, it is likely that 1,25(OH)_2_D and the VDR influence skeletal muscle via both genomic and non-genomic mechanisms (Schmidt et al., 2000; Nguyen et al., 2004;).

### Genomic actions of vitamin D

Typical of steroid hormones, the binding of 1,25(OH)_2_D to the VDR results in enhanced transcription of a range of proteins, including those involved in calcium metabolism (Fleet, 2004). Calcium is a critical modulator of skeletal muscle function and any perturbation to calcium handling may impact on both its contractile and relaxation properties (Berchtold et al., 2000). Therefore, 1,25(OH)_2_D may affect muscle function through both calcium-related protein transcription and total body calcium levels. Recently, however, it has transpired that 1,25(OH)_2_D also has a transcription-enhancing role on proteins other than those involved directly in calcium metabolism. One such protein, relevant to the discussion of skeletal muscle, is IGFBP-3. In order to illustrate the potential impact of 1,25(OH)_2_D on skeletal muscle function, the role of insulin-like growth factor-1 (IGF-1) and IGFBP-3 will be described in further detail.

IGF-1 is a 7.5 kDa polypeptide, structurally similar to insulin (Thissen et al., 1994). It induces proliferation, differentiation and hypertrophy of skeletal muscle (Barton-Davis et al., 1998) and is a key component in muscle regeneration (Schertzer et al., 2007). IGF-1 has at least three isoforms resulting from splice variations, namely IGF-1Ea, IGF-1Eb and IGF-1Ec (Goldspink, 2005). IGF-1Ea is the circulating form of IGF-1 expressed from the liver, whereas IGF-1Ec, also known as mechano-growth factor (MGF), is the tissue isoform released from skeletal muscle cells, believed to exert exclusively autocrine/paracrine actions (Goldspink, 1999). Each isoform may have slightly different biological actions, with IGF-1Ea stimulating terminal differentiation of muscle cells into myotubes and promoting stem-cell mediated muscle regeneration. By contrast, MGF responds to tissue damage, controls local tissue repair and is more potent than IGF-1Ea at causing hypertrophy (Goldspink, 1999). Subsequently, IGF-1 is recognized as both a potential means for addressing age-related sarcopenia (Grounds, 2002) and as an illegal ergogenic aid in sport (Adams, 2000). IGF-1 circulates in the serum 99% bound to a carrier protein IGFBP-3. Only 1% of serum IGF-1 is “free” (fIGF-1) to exert the biological effects, and when unbound, IGF-1 is rapidly cleared (Liao et al., 2008).

**Fig. 1:**
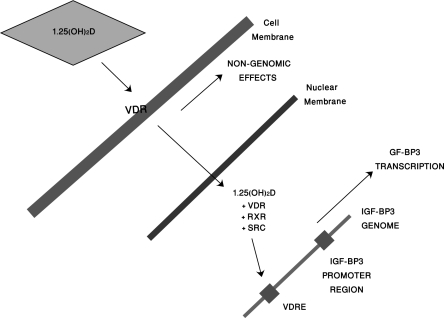
Potential role for 1,25-OH vitamin D in non-calcium-related, genomic action in muscle cells.

IGFBP-3 is a member of the IGFBP family, which bind IGF-1 in the serum, the extracellular matrix or on cell surfaces (Berg et al., 2007) with high affinity and specificity (Baxter, 2000; Peng et al., 2004;). The binding of IGF-1 to IGFBP's may have both inhibitory and stimulatory effects on IGF-1 function (Baxter, 2000). The IGF-1–IGFBP-3 complex may block the binding of IGF-1 to its receptors, thereby mitigating its effect on DNA synthesis, growth and glucose regulation (Baxter, 2000), but preventing its otherwise rapid clearance (Liao et al., 2008). Furthermore, presumably as a result of increased free IGF-1, the inhibition of IGF-1 binding to IGF-1BP has been shown to enhance the healing of mice skeletal muscle (Schertzer et al., 2007). It has also been suggested that IGFBP may have a role independent of any IGF-1 binding, inhibiting both DNA synthesis and inducing apoptosis (Baxter, 2000; Peng et al., 2004;). IGFBP-3 expression is regulated by a number of factors, including 1,25(OH)_2_D (Peng et al., 2004), with a VDRE in the promoter region for human IGFBP-3 recently identified (Peng et al., 2004). Thus, 1,25(OH)_2_D, in combination with the VDR and other elements, such as SRC-3 (Liao et al., 2008), can positively influence IGFBP-3 expression (Fig. 1). For example, without SRC-3 cofactor combination with the activated VDR, transcriptional expression of IGFBP-3 is reduced, and as a result IGF-1 clearance is increased (Liao et al., 2008).

The potential significance of this process involving IGF-1 was illustrated in a recent investigation of children with vitamin D-deficient rickets, before and after supplementation with vitamin D (Soliman et al., 2008). The authors found that the growth rates and height of the children increased with vitamin D supplementation, and that there was a significant correlation between serum concentrations of IGF-1 and the percentage increment in 25(OH)D concentrations, with IGF-1 concentrations increasing significantly after treatment with vitamin D. Both the length and the growth rate correlated with the IGF-1 concentration, leading the authors to conclude that the growth spurt observed in children with rickets after vitamin D supplementation is mediated via through an increase in IGF-1 (Soliman et al., 2008).

### VDR polymorphisms and skeletal muscle function

The VDR gene located on chromosome 12 (12q13.11) (Bray et al., 2009) is known to have various genetic polymorphisms including Bsm1, Fok1, Apal and Taq1, which have been associated with various functional outcomes (Geusens et al., 1997; Roth et al., 2004; Hopkinson et al., 2008;). The Fok1 polymorphism involves a T to C transition in exon 2 of the VDR gene, resulting in a shorter (424) amino acid VDR than the T allele (427) (Guo et al., 2006), and has been associated with variations in both bone mineral density (Zhang et al., 2008) and differential responses of bone density to strength training (Tajima et al., 2000; Rabon-Stith et al., 2005;). Furthermore, an association with fat-free mass and risk of age-related sacropenia has been described (Roth et al., 2004) and in patients suffering from chronic obstructive pulmonary disease (COPD), Fok1 C homozygotes (also known as FF) were found to have significantly weaker quadriceps than either CT heterozygotes or T (ff) homozygotes (Hopkinson et al., 2008).

Geusens et al. (1997) assessed the impact of VDR polymorphis ms on grip and quadriceps strength. Five hundred and one healthy women over the age of 70 were assessed for quadriceps and grip strength and the VDR genotype Bsm1 (a single nucleotide polymorphism found in intron 8 (Guo et al., 2006)). In this cross-sectional study, the bb genotype (that is the presence of the restriction site on both alleles) was found to be significantly stronger than BB or heterozygote genotype. This finding was supported by a study involving patients with COPD, whereby the bb polymorphism was associated with stronger quadriceps muscles (Hopkinson et al., 2008). However, not all studies have shown similar results. Grundberg et al. (2004) examined the relationship between Bsm1 polymorphisms and muscle strength utilizing 170 pre-menopausal Swedish women. Women homozygous for Bsm1 BB or poly-A repeat ss were found to have higher hamstring strength than the bb or LL genotypes. Furthermore, no significant associations were found between VDR polymorphisms and either grip strength or quadriceps strength. Similarly, Roth et al. (2004) found no impact of the Bsm1 polymorphism on fat-free mass or sarcopenia in elderly men.

### Non-genomic effects of vitamin D on muscle

A pathway of vitamin D action, independent of the intra-nuclear transcription process, was mooted in the 1980s and has been characterized more recently (Boland et al., 1995; Nguyen et al., 2004;). 1,25(OH)_2_D has been shown to be involved in the rapid regulation of membrane calcium channels in cultured chick skeletal muscle cells (Vazquez et al., 1997). Subsequently, a membrane receptor in rat chondrocytes with a higher molecular weight than the intra-nuclear VDR (known as the membrane-associated rapid response steroid-binding protein (MARRS) (Fleet, 2004)), specific for 1,25(OH)_2_D, has been identified (Nemere et al., 1998). Recent evidence illustrating that the application of 1,25(OH)_2_D results in the translocation of the VDR to the plasma membrane in chick skeletal muscle cells (Capiati et al., 2002) and that the rapid effects of vitamin D require the VDR (Nguyen et al., 2004) suggests that a combination of both the intra-nuclear VDR and other membrane receptors (i.e., MARRS) may be involved in the rapid actions of vitamin D (Capiati et al., 2002). While the exact mechanism of the non-genomic action of vitamin D remains a controversial and heavily researched topic, it is widely accepted that vitamin D levels have a rapid effect on the membrane calcium channels of muscle cells in numerous species (Schmidt et al., 2000; Fleet, 2004; Nguyen et al., 2004;). As calcium is a critical modulator of skeletal muscle function (Berchtold et al., 2000), it follows that vitamin D levels may have a significant impact on muscle function, performance and injury risk.

## Conclusion

Vitamin D deficiency has traditionally been considered the domain of the elderly; however, this is changing as evidence of high rates of vitamin D deficiency is recognized in today's youth. Vitamin D deficiency is now endemic in many communities and athletes are not spared this condition. Increasingly, vitamin D deficiency is recognized as being associated with both chronic health conditions and musculo-skeletal injuries such as stress fractures; however, its potential impact on other tissues such as muscle is not well described.

The identification of the VDR, its various polymorphisms, and variable expression with aging, has provided some insight into the complex mechanisms by which vitamin D and its metabolic pathways may affect muscle function. The recognition of both genomic and non-genomic effects of vitamin D in skeletal muscle, with the resultant impact on both calcium metabolism and protein transcription, further illustrates the significance of vitamin D in muscle function. Further clarification of the complex role of the VDR in both muscle and other musculo-skeletal tissues is required.

Despite the limited evidence available at the time, athletes and trainers in the early 20th century believed that UVB radiation was beneficial to athletic performance. Accumulating evidence supports the existence of a functional role for vitamin D in skeletal muscle with potentially significant impacts on both the performance and injury profiles of young, otherwise healthy athletes. While further research is required to evaluate the level of vitamin D required for optimal muscular function, clinicians working with athletes should be aware of the broad impact of vitamin D deficiency on the athlete.

Vitamin D should no longer be considered only to have an impact on calcium metabolism and bone morphology, but should be recognized to have a broad impact on the organism as a whole. Further epidemiological and *in vitro* research into the potential impact of vitamin D deficiency on muscle function, morphology and performance in young athletic individuals is required.
